# 

**Published:** 1884-04

**Authors:** 


### BOOKS AND PAMPHLETS RECEIVED.

14TH ANNUAL REPORT OF THE MANHATTAN EYE AND EAR HOSPITAL, NEW YORK.

ANNOUNCEMENT, NEW YORK POST-GRADUATE MEDICAL SCHOOL, 213-215 East 23d Street, session 1883–4.

ANNUAL ADDRESS DELIVERED BEFORE THE AMERICAN ACADEMY OF MEDICINE, by Henry O. Marcy, A. M., M. D.

THE HORSE'S FOOT AND ITS DISEASES, BY A. ZUNDEL. Translated by A. Liautard, M. D. V. S., Professor of Anatomy and Operative Surgery, American Veterinary College. 8vo, pp. 167. New York: W. R. Jenkins, 1884.

JOHNS HOPKINS UNIVERSITY CIRCULARS.

ANNALS ET BULLETIN DE LA SOCIÉTIÉ DE MEDICINE DE GAND.

JOURNALS.—La Clinica Veterinaria, Milano. The Veterinarian, London; The Veterinary Journal, London; Der Zoologische Garten, Frankfort; Schweizerisches Archiv fur Thierheilkunde, Zurich; Archiv fur Wissenschaftliche und Praktische Thierheilkunde, Berlin; Journal de la Société contre L'Abus du Tabac, Paris; Tidskrift for Veterinar Medicine, Stockholm; Giornale di Anatomia, Fisiologia e Patologia, degli Animali, Pisa; Il Medico Veterinario, Torino; El Ensayo Medico, Caracas; Kansas City Review of Science and Industry; Chicago Medical Journal and Examiner; The Medical Record, New York; The College and Clinical Record, Philadelphia; New England Medical Monthly, Sandy Hook; Chicago Medical Review; Virginia Medical Monthly, Richmond; Nashville Journal of Medicine and Surgery; The Southern Clinic, Richmond; The Western Medical Reporter, Chicago; Journal of Cutaneous and Venereal Diseases, New York; Denver Medical Times; The St. Joseph Medical Herald; The Weekly Medical Review, St. Louis; The Blacksmith and Wheelwright, New York; National Live Stock Journal, Chicago; The Breeder's Gazette, Chicago; Cultivator and Country Gentleman, Albany; Weekly Drover's Journal, Chicago; The Chicago Tribune; Spirit of the Times, New York; United States Veterinary Journal, Chicago; Horseshoer and Hardware Journal, Chicago; The Polyclinic, Philadelphia; Medical World, Philadelphia; The New York Medical Journal; American Veterinary Review, edited by Prof. A. Liautard, H. F. R. C. V. S., New York; Atlantic Journal of Medicine, Richmond; Mirror of American Sports, Chicago; San Francisco Western Lancet; La Cronica Medica, Valencia.

